# Transition to suicide ideation and attempt among emergency room patients

**DOI:** 10.1192/bjo.2024.24

**Published:** 2024-04-18

**Authors:** Claudio Yurdadön, Ronny Bruffaerts, Marc Sabbe, Koen Demyttenaere, Elke Peeters, Franco Gericke, Wouter Voorspoels

**Affiliations:** KU Leuven, Leuven, Belgium; Department Medical Affairs, Janssen-Cilag NV, Beerse, Belgium; KU Leuven, Leuven, Belgium; and UZ Leuven, Leuven, Belgium

**Keywords:** Suicidal thoughts and behaviours, depression, primary care, psychiatric emergency room, public health

## Abstract

**Background:**

Suicidal thoughts and behaviours (STB) represent a persistent and serious public health problem, and suicide is among the leading causes of death worldwide. We focus on predictors of transition rates and time courses through the STB spectrum among psychiatric emergency room (PER) patients.

**Aims:**

We aimed to investigate (a) whether currently suicidal patients had prior referrals to the PER, (b) for which reason they were previously referred to the PER and (c) the timing of this referral.

**Method:**

We performed a retrospective study spanning 20 years with 24 815 PER referrals. Descriptive statistics of patients’ sociodemographic and clinical characteristics are provided and expressed as weighted proportions and means. Logistic regression was used to identify risk profiles of patients who had a higher chance of being referred for reasons of STB given their PER history. Multiple imputation and data weighting techniques were implemented.

**Results:**

STB among PER patients was persistent and led to repeated referrals (up to five times more likely), often within a short period (18% <1 month). Those previously referred for ideation/plan had 66% higher risk of making the transition to suicide attempt, with 25% making this transition within a month after previous referral. This is similar to the transition from depressed mood to suicide ideation/plan.

**Conclusions:**

STBs in PER patients are persistent and lead to repeated referrals, often within a short period, including transitions to more severe forms of STB.

Suicidal thoughts and behaviours (STB) represent a persistent and serious public health problem,^1^ and suicide is among the leading causes of death worldwide.^2^ Although prevention programmes and psychological treatments have been established over the years, STB patients are generally reluctant to seek help. Worldwide estimates show 12-month treatment rates of approximately 4 in 10 for people who have engaged in any suicidal behaviour (ideation, plan or attempt),^3^ with estimates as low as 40% of patients with a suicide attempt.^4^ Notably, among those with STB that do receive treatment, the majority do not seek treatment from specialised services but instead in low-threshold sectors such as primary care and, perhaps more importantly, in emergency rooms.^5,6^ STB is estimated to make up approximately 10% of all referrals to the emergency room.^7–9^ Furthermore, there is evidence that patients who present at hospitals with STB are prone to repeated visits for similar reasons.^10^

Previous studies have made detailed records of the prevalence of STB^11–13^ but have generally been unable to describe transitions over time (see Griffin et al^10^ and Owens et al^14^ for notable exceptions). STB is a persistent problem, with roughly two-thirds experiencing some form of STB up to 10–20 years after the first occurrence.^15^ Studies on the time course of STB in the emergency room are inconclusive, mainly owing to limited sample sizes.^16^ Thus, surprisingly little is known about the time course of the transitions patients make within the suicidal spectrum.

In this study, we focus on transition rates and the associated time frame through the STB spectrum. In addition, we highlight the strength of the associations between a broad spectrum of symptoms for referral in the past and current STB among psychiatric emergency room (PER) patients. Based on PER data from the Leuven Study of Emergency Psychiatry,^17^ which now span 20 years, we aimed to estimate how many current suicidal patients had prior referrals to the PER, the reasons they were previously referred and the timing of this referral.

## Method

### Setting

The study was conducted at the University Hospital Gasthuisberg in Leuven, Belgium. Leuven has nearly 100 000 inhabitants, and the hospital catchment area includes approximately 250 000 people. The university hospital is the only public healthcare service with a psychiatric emergency team. Patients visiting the hospital emergency room with potential psychiatric complaints are automatically followed up by a psychiatric emergency team. The long-standing collaboration between the emergency room and PER has raised general awareness of patients with psychiatric problems, with a focus on detecting STB, in the context of the high suicide rates in Belgium.^18,19^ A full range of emergency evaluation, intervention, referral and disposition services are provided for adult patients in crisis, 24 h a day, at the university hospital's emergency room.

### Data collection

Institutional ethics committee approval was obtained for this study (ethics committee of UZ Leuven, KU Leuven, S64103). All methods were carried out in accordance with relevant guidelines and regulations (declarations of Helsinki). The ethics committee of UZ Leuven exempted informed consent as all information that could identify a patient in relation to their clinical status or identity have been removed and anonymised.

Trained staff members of the PER evaluated patients using structured assessment instruments. A semi-structured interview based on the Minimal Psychiatric Data form, a standardised and validated psychiatric patient registration form that has been used by the Belgian Ministry of Social Affairs, Public Health, and Environment since 2000,^17,20–21^ was administered to gather information about patients’ demographic characteristics, patterns of referral, clinical characteristics (reasons for referral), and past and present mental health service use, including previous admissions to the PER, as well as whether they had been referred elsewhere following the PER visit. The registration form for the PER semi-structured interview is intended to be concise and structured, given the PER context.

This is a retrospective study on clinical data spanning almost two decades; all information that could identify a patient in relation to their clinical status had been removed and anonymised; therefore, informed consent was not needed. Owing to changes in personnel^a^, hospital guidelines and peak occupancy at the PER, not every patient visit to the PER in the time frame of 2000–2020 is recorded in the database. There is no reason to assume any selective bias in our sample (non-registration was random), but nonetheless we have added post-stratification weights on the basis of gender and age to our analyses.

#### Symptoms for referral

Upon arrival in the PER, a standardised set of information was collected from the patient on the basis of a semi-structured interview, including their clinical profile and information about any previous referrals to the PER. Clinical characteristics of referred patients were assessed in terms of their presented symptomatology (in what follows: reason for referral) by a psychiatrist and classified into a number of robust categories:^3,17^ suicide attempt, suicide ideation (including plans), aggression and/or violence towards others, anxiety symptoms, mood symptoms, substance misuse, psychotic symptoms, symptoms caused by an underlying physical condition (such as brain tumours, or neurological or endocrine disorders) and other problems. In the semi-structured interview, suicide attempt was coded only in case of a clear suicidal intent, no distinction was made between suicide ideation and plan, and non-suicidal self-injury (NSSI) was not included in the registration form as a separate category. Each referral in the database was termed a current referral and associated with a reason for the current referral. If a patient (the current referral) indicated having been in the PER before, a previous referral was recorded and associated with a reason for the previous referral. In addition, the time between the previous and current referral was categorised as follow: <1 month, 1–3 months, 3–6 months, 7–12 months or >12 months.

### Statistical procedures

For all analyses, missing data of key variables such as mental health status and history (reason for referral, reason for previous referral) were multiply imputed by chained equations,^22^ simulating ten data-sets using a maximum of 30 iterations. The quality of the imputation was assessed by visual inspection of convergence and comparison of imputed and observed variable distributions. To ensure the representativity of the PER sample, post-stratification weights for gender and age were used in all analyses, on the basis of descriptive statistics of the PER population in the same time frame.

Descriptive statistics of the patients’ sociodemographic and clinical characteristics are provided as counts and proportions, weighted for gender and age to match the population of PER referrals in the hospital in the same time frame, and presented with the appropriately adjusted confidence intervals. Chi-squared tests were used to compare the demographic characteristics of patients with and without previous referral to the PER.

To sketch the pathways within the STB spectrum across sequential referrals, we calculated conditional proportions (i.e. the proportion of those who were referred previously, e.g. for STB). To evaluate the statistical significance and strength of the associations between reasons of previous referral (see section ‘Symptoms for referral’) and the current referral, two logistic regression analyses were performed, one with current suicide ideation and/or plan as outcome and one with suicide attempt as an outcome, and the reasons for the previous referral as predictors. Sociodemographic characteristics are also included as covariates in the model. The strength of association between predictors and outcome is expressed in terms of adjusted odds ratios (AOR) presented with 95% confidence intervals. In the analyses presented, the baseline category for comparison of odds was those patients who had never previously been referred to the PER. As such, we used all data in the logistic regressions. This was a reasonable choice as it enabled us to compare the odds of a particular outcome (e.g. suicide attempt) in a target category (e.g. those with a previous referral for suicide ideation/plan) with the odds of those without a previous referral to PER. The odds ratio thus effectively compared the odds of the outcome in the target category with the base rate. In a sensitivity analysis, we repeated the same analysis, focusing exclusively on those patients with a previous referral to PER. The results were qualitatively identical.

Associations between the reason for previous referral and the timing (a categorical variable, see above) of the current referral were assessed using Pearson's chi-squared tests. All analyses were performed in R version 4.1.0.^23^ For multiple imputation, we used the ‘mice’ package;^22^ for calibration weighting and inferential statistics, we used the ‘survey’ package.^24^ All analysis scripts can be found here at https://osf.io/hzg6v/.

## Results

### Sample description

The sample from the UZ Leuven hospital included *N* = 24 815 patient referrals of adults (aged 18 years and older), between the years 2000 and 2020. Table 1 shows the demographic characteristics of the PER population (all referrals) between 2000 and 2020. Of all referrals, 51.7% (95% CI: 51.1–52.3%) were female; 59.6% (95% CI: 59–60.3%) were living together and 31.8% (95% CI: 31.2–32.4%) were employed. The mean age was 41.8 years (s.d. = 15.5). Looking at the reasons for referral, 23.8% (95% CI: 22.7–25.0%) were referred because of substance misuse, 16.0% (95% CI: 15.2–16.7%) for depressive symptomology, 14.2% (95% CI: 13.6–14.9%) owing to suicide ideation/plan, 9.5% (95% CI: 7.4–11.6%) for suicide attempts and 8.6% (95% CI: 8.1–9%) for psychotic symptoms.

**Table 1:** Sociodemographic characteristics for PER referrals, with and without a previous referral to the PER

Attribute	Category	All referrals		Previous PER referrals		No previous PER referral	
		*N*	Weighted % (95% CI)	*N*	Weighted % (95% CI)	*N*	Weighted % (95% CI)
Gender**	Female	12 824	51.7% (51.1–52.3%)	3179	49% (47.8–50.2%)	9645	52.6% (51.9–53.3%)
	Male	11 991	48.3% (47.7–48.9%)	3302	51% (49.8–52.2%)	8689	47.4% (46.7–48.1%)
Age, years**	35–49	8950	30.7% (30.1–31.2%)	2558	34.4% (33.2–35.5%)	6392	29.4% (28.7–30%)
	25–34	5162	21% (20.5–21.5%)	1452	22.9% (21.9–23.9%)	3710	20.3% (19.7–20.9%)
	50–64	4816	21.7% (21.2–22.2%)	1252	21.9% (20.8–22.9%)	3564	21.7% (21–22.3%)
	18–24	3899	16.3% (15.8–16.8%)	938	15.3% (14.4–16.2%)	2961	16.7% (16.1–17.2%)
	65–75	1047	5.3% (5–5.6%)	205	4% (3.5–4.6%)	842	5.7% (5.4–6.1%)
	76–100	941	5% (4.7–5.4%)	76	1.6% (1.2–1.9%)	865	6.2% (5.8–6.6%)
Living arrangements**	Together	12 915	59.6% (59–60.3%)	3162	49.6% (48.4–50.9%)	9753	63.1% (62.4–63.9%)
	Alone	8042	36.1% (35.5–36.7%)	2859	45% (43.8–46.3%)	5183	33% (32.3–33.7%)
	In therapy	462	2.1% (1.9–2.3%)	151	2.4% (2–2.8%)	311	2% (1.8–2.2%)
	Unknown	442	2% (1.8–2.1%)	161	2.5% (2.1–2.9%)	281	1.8% (1.6–2%)
	No residence	47	0.2% (0.1–0.3%)	24	0.4% (0.2–0.5%)	23	0.1% (0.1–0.2%)
Working arrangements**	employed	6825	31.8% (31.2–32.4%)	1595	24.8% (23.7–25.9%)	5230	34.2% (33.5–34.9%)
	Disabled	5837	25.4% (24.9–26%)	2256	34.9% (33.8–36.1%)	3581	22.1% (21.5–22.8%)
	Unemployed	4240	18.9% (18.4–19.5%)	1440	22.8% (21.7–23.8%)	2800	17.6% (17–18.2%)
	Retired	2285	12.5% (12–12.9%)	407	7.8% (7.1–8.6%)	1878	14.1% (13.5–14.7%)
	Unknown	1026	4.7% (4.5–5%)	332	5.4% (4.8–6%)	694	4.5% (4.2–4.8%)
	Student	1007	4.8% (4.6–5.1%)	174	3.1% (2.6–3.5%)	833	5.5% (5.1–5.8%)
	Housewife	361	1.8% (1.6–2%)	76	1.2% (0.9–1.5%)	285	2% (1.7–2.2%)

Chi-squared test was used to compare those with a previous referral to the PER with those without a previous referral.

** *P* < 0.01.

Among the subgroup of patients who had had a previous referral to the PER (*N* = 6481, approximately 26%), 49.0% (95% CI: 47.8–50.2%) were female, 24.8% (95% CI: 23.7–25.9%) were employed and 45.0% (95% CI: 43.8–46.3%) were living alone. The mean age was 40.5 years (s.d. = 13.3). More detailed comparisons of this subgroup with the patients who did not have a previous referral suggested that the subgroup of patients with a previous referral was distinct in terms of demographics (Table 1) and clinical characteristics (Table 2) Comparing those with a referral history with those without, patients with a previous referral were more likely to be male (51%, χ²(1, *N* = 24 815) = 25.02, *P* < 0.001), were generally younger (34.4% *v.* 30.7% in the 35–49 age group, χ²(5, *N* = 24 815), *P* < 0.001), and were more often living alone (45% *v.* 36.1%, χ²(1, *N* = 24 815) = 306.04, *P* < 0.001) and without a job (22.8% *v.* 18.9, χ²(1, *N* = 24 815) = 186.2, *P* < 0.001).

**Table 2:** Reasons for referral among all PER patients, those with a previous referral and those without a previous referral

Reason for referral	All referrals		Previous PER referrals		No previous PER referral	
	*N*	Weighted % (95% CI)	*N*	Weighted % (95% CI)	*N*	Weighted % (95% CI)
Substance use*	5917	23.8% (22.7–25%)	2064	32.3% (30.8–33.7%)	3854	20.9% (19.5–22.3%)
Depressive symptoms**	3967	16% (15.2–16.7%)	819	12.8% (11.7–13.9%)	3149	17.1% (16.2–18%)
Suicide ideation/plan	3535	14.2% (13.6–14.9%)	883	13.8% (12.8–14.8%)	2652	14.4% (13.7–15.1%)
Other**	3567	14.4% (13.5–15.2%)	804	12.6% (11.7–13.5%)	2762	15% (13.9–16.1%)
Suicide attempt	2352	9.5% (7.4–11.6%)	606	9.5% (7.4–11.5%)	1746	9.5% (7.3–11.7%)
Psychotic symptoms*	2129	8.6% (8.1–9%)	494	7.7% (7–8.4%)	1635	8.9% (8.3–9.5%)
Aggression/violence towards others	1183	4.8% (4.5–5.1%)	310	4.9% (4.2–5.5%)	873	4.7% (4.4–5.1%)
Symptoms caused by physical condition*	1033	4.2% (3.8–4.5%)	212	3.3% (2.8–3.8%)	822	4.5% (4.1–4.9%)
Anxiety**	656	2.6% (2.4–2.9%)	124	1.9% (1.5–2.4%)	532	2.9% (2.6–3.2%)
Agitation/confusion**	474	1.9% (1.6–2.2%)	74	1.2% (0.9–1.5%)	400	2.2% (1.8–2.5%)

Chi-squared test was used to compare those with a previous referral to the PER with those without a previous referral.

*P < 0.05, **P < 0.01.

PER, psychiatric emergency room.

As shown in Table 2, the subgroup of patients with a previous referral to PER was also different from the general PER patient group with regard to reasons for current referral to the PER (χ²(9, *N* = 24 815) = 15.112, *P* < 0.001). Notably, more patients with a previous referral had substance use problems (32.3% *v.* 23.8%, χ²(1, *N* = 24 815) = 99, *P* < 0.001), and fewer patients with a previous referral had depressive symptoms (11.5% *v.* 17.1%, χ²(1, *N* = 24 815) = 85.3, *P* < 0.001) compared with patients without a previous referral.

### Suicide ideation/plan and suicide attempt at present and previous PER referral

**Fig. 1:**
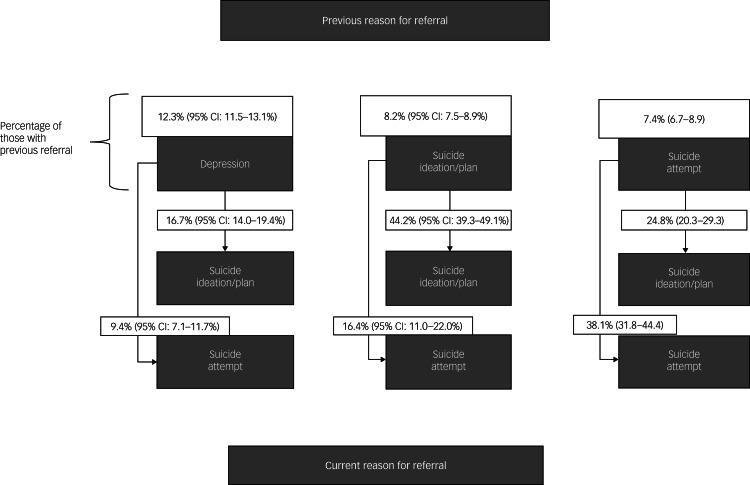
Pathways within the STB spectrum. The top layer shows the three most relevant reasons for previous referral associated with a current referral for STB, with the percentages (and 95% confidence intervals) on top of the box indicating their respective proportions. The arrows indicate the pathways and the proportion of patients who move from a previous referral to current suicide ideation/plan or suicide attempt. STB, suicidal thoughts and behaviours.

In total, 23.3% (95% CI: 21.7–24.9%) of patients with a previous referral were currently referred for STB. In this section, we sketch out the pathways within the STB spectrum in more detail. The diagram in Fig. 1 summarises the main tendencies of those individuals who had been previously referred for depressive symptoms or STB and were currently readmitted for reasons of STB.

#### Pathways to and in STB in the PER

##### Readmission for suicide ideation/plan

Of all patients with a previous referral to the PER, 18.1% (95% CI: 17.0–19.2%) had been readmitted for current suicide ideation/plan. Of these patients, the majority had been previously referred to the PER for suicide ideation/plan (23.4%, 95% CI: 20.8–26.0%), followed by substance misuse (23.7%, 95% CI: 20.9–26.4%) and depressive symptoms (15.6%, 95% CI: 13.5–17.8%).

Considering the pathways to suicide ideation/plan from previous reasons for referral, the persistence of suicide ideation/plan was most prominent: for 44.2% (95% CI: 39.3–49.1%) of patients who indicated they had been in the PER previously for suicide ideation/plan, the reason for the current referral was also suicide ideation/plan (Fig. 1). The second most prominent pathway was from suicide attempt to suicide ideation and/or plan (24.8%, 95% CI: 20.3–29.3%). Third, 16.7% (95% CI: 14.0–19.4%) of patients previously referred for depressed mood had been readmitted for suicide ideation/plan. Pathways from other reasons for previous referral were substantially less frequent (e.g. only 6.4%, 95% CI: 5.2–7.6%) previously referred for substance misuse problems were readmitted for suicide ideation/plan).

##### Readmission for suicide attempt

Of patients with a previous referral to the PER, 9.72% (95% CI: 7.7–11.8%) had been readmitted for suicide attempt (i.e. current referral). For these patients, the most prevalent reason for the previous referral was suicide attempt (29.0%, 95% CI: 22.0–36.0%), followed by substance misuse (21.4%, 95% CI: 14.0–28.8%) and suicide ideation/plan (15.6%, 95% CI: 13.5–17.8%).

Similar to suicide ideation/plan, suicide attempt was very persistent across subsequent referrals to the PER. As shown in Fig. 1, for 38.1% (95% CI: 31.2–44.2%) of patients who indicated that they had been in the PER previously for suicidal attempt, this was also the reason for the current referral. The second most frequent pathway was from suicide ideation/plan to attempt; 16.48% (95% CI: 11–22%) previously referred for suicide ideation/plan had been readmitted for suicide attempts. Other reasons for previous referral appeared to be less associated with a current referral for suicide attempt; e.g. only 9.4% (95% CI: 7.1–11.7%) of those previously referred for substance misuse and 4.81% (95% CI: 2.0–7.6%) of those with previous substance misuse problems had been readmitted for suicide attempt.

### Multivariate predictors of readmission to suicide ideation/plan and timing across PER referrals

We assessed risk factors for suicide ideation/plan using logistic regression. All predictors (in addition to age and gender) are shown in Table 3. The fitted model had an area under the curve of 0.62. Compared with those who had no previous referral to the PER, the most powerful predictors of a referral for suicide ideation/plan were: previous referral for suicide ideation/plan (AOR = 4.17, 95% CI: [3.43–5.08]), previous referral for suicide attempt (AOR = 1.64, 95% CI: [1.30–2.07]) and previous referral for depressive symptomology (AOR = 1.33, 95% CI: [1.12–1.59]). The odds of referral for suicide ideation/plan were smaller for those previously referred to the PER for substance misuse reasons (AOR = 0.55, 95% CI: [0.47–0.63]). Further analysis on this point revealed that substance use was persistent across sequential referral to the PER. Patients with a previous referral to PER for substance use were more likely to be currently referred for substance use again (AOR = 5.23, 95% CI: [4.68–5.84]); this may well have overwhelmed potential associations with STB. Table 3 shows the association between reasons for previous referral and current suicide ideation/plan, and the timing of these readmissions.

**Table 3:** AOR and 95% CI values for predictors of a current referral for suicide ideation/plan and proportions of timings to readmission

Previous reason	AOR^a^ (95% CI)	<1 month	1–3 months	4–6 months	7–12 months	>1 year
Suicide ideation/plan**	4.17 (3.43–5.08)**	34.67	15.99	12.44	10.82	26.08
Suicide attempt**	1.64 (1.30–2.07)**	21.37	20.20	9.83	12.28	36.33
Depressive symptoms**	1.33 (1.12–1.59)**	22.06	14.60	12.98	11.19	39.17
Unknown	1.25 (1.00–1.56)	17.94	16.01	14.21	16.85	34.99
Other*	0.72 (0.55–0.94)*	26.79	11.28	14.60	14.01	33.32
Substance use**	0.55 (0.47–0.63)**	22.24	14.13	14.46	10.68	38.48
Anxiety	0.50 (0.19–1.29)	5.28	0.00	20.31	19.09	55.32
Agitation/confusion	0.44 (0.10–1.96)	0.00	0.00	63.00	27.55	9.44
Psychotic symptoms**	0.43 (0.30–0.63)**	15.49	16.79	14.92	16.67	36.13
Aggression/violence towards others*	0.42 (0.22–0.80)*	21.37	19.11	18.92	0.60	39.99
Symptoms caused by physical condition*	0.27 (0.08–0.90)*	0.00	31.03	58.83	0.00	10.14

a. All AORs are adjusted for age, gender and all other predictors in the table.

*P < 0.05, **P < 0.01.

AOR, adjusted odds ratios.

Approximately half of patients currently referred for ideation/plan who had been previously referred for ideation/plan were referred to the PER again within 3 months and one-third within a month. Of those previously referred because of a suicide attempt, approximately 38% were referred to the PER for suicide ideation/plan within 3 months. Similarly, 36.0% of those with previous referrals for depressive symptoms were readmitted for suicide ideation/plan within 3 months. A chi-squared test provided no evidence for an association between the previous reason for referral and the timing of the transition to a current referral for suicide ideation/plan to the PER (χ²(28, *N* = 3535) = 58.11, *P* = 0.165).

### Multivariate predictors of readmission for suicide attempt and timing across PER referrals

Results from the logistic regression analysis for suicide attempt at current referral were similar. The fitted model had an area under the curve of 0.61. Compared with those without previous history of referral to the PER, the most powerful predictors of a referral for suicide attempts were previous referral for suicide attempt (AOR = 5.34, 95% CI: [3.61–7.89]) and previous referral for ideation/plan (AOR = 1.66, 95% CI: [1.06–2.61]). Conversely, the odds were lower for those previously referred for substance use issues (AOR = 0.52, 95% CI: [0.38–0.72]).

Table 4 shows the timings of these readmissions for suicidal attempt. Approximately a quarter of those referred currently for suicide attempt and with a previous referral for ideation/plan were readmitted within 1 month, and half within the first 3 months since their previous referral. Notably, almost half of those repeated suicide attempt patients repeated their attempt within 6 months. Similar trends for previous reasons of depressive symptoms and substance misuse are shown in Table 4. A chi-squared test provided no evidence for an association between the previous reason for referral and the timing of the transition to the current referral for suicide attempt to the PER (χ²(28, *N* = 2352) = 54.99, *P* = 0.93).

**Table 4:** AOR and 95% CI values for predictors of a current referral for suicide attempts and proportions of timings to readmission

Previous reason	AOR^a^ (95% CI)	<1 month	1–3 months	4–6 months	7–12 months	>1 year
Suicide attempt**	5.34 (3.61–7.89)*	18.12	19.66	9.07	12.62	40.53
Suicide ideation/plan*	1.66 (1.06–2.61)*	25.43	20.93	13.74	13.35	26.55
Unknown	1.01 (0.76–1.35)	26.78	15.85	11.36	10.02	35.99
Depressive symptoms	0.91 (0.68–1.21)	18.86	15.37	11.55	18.71	35.50
Anxiety	0.79 (0.24–2.66)	30.42	0.00	57.66	3.29	8.63
Aggression/violence towards others	0.64 (0.30–1.35)	14.99	28.95	28.17	14.69	13.19
Other*	0.58 (0.39–0.86)*	20.66	15.74	15.97	9.14	38.50
Psychotic symptoms*	0.55 (0.35–0.86)*	16.23	13.56	10.81	18.93	40.46
Substance use*	0.52 (0.38–0.72)*	16.61	14.94	14.13	12.01	42.31
Symptoms caused by a physical condition	0.15 (0.02–1.15)	0.00	0.00	5.29	0.00	94.71

a. All AORs are adjusted for age, gender and all predictors in the table.

*P < 0.05, **P < 0.01.

AOR, adjusted odds ratios.

### Sensitivity analysis

For both models, a sensitivity analysis revealed that the results were robust against modelling choices. First, we restricted the analyses to referrals with a previous referral to the PER. In addition, we ran the different versions of the analyses leaving out certain categories to check for potential contamination of the results: the categories ‘unknown’, ‘others’ and ‘medical reasons’ were progressively left out. In all cases, the odds ratios reported for depressive mood, suicide ideation/plan and suicide attempt were quantitatively similar and in no instance did conclusions qualitatively change.

## Discussion

In this study, we examined the proportion, predictors and timing of readmissions for STB in patients referred to the PER. Four main findings stand out: (a) 18.1% of patients with previous referrals were readmitted for suicide ideation/plan and 9.7% for suicide attempt; (b) there was high persistence in STB across PER referrals (38–44%); (c) being previously referred for depressed mood was associated with readmission for suicide ideation/plan; and (d) for 19–35% of the patients with previous referrals for STB or depressed mood, the time between referrals was less than 1 month.

In line with previous reports, substance misuse and STB were common clinical reasons for referrals among patients with multiple referrals^25^ and coexisted in PER patients.^26^ Against the scarcity of knowledge of the suicidal process,^27^ there was a strong temporal association between suicide ideation/plan and suicide attempt, with respect to which four findings warrant attention. First, STB among PER patients was persistent and led to repeated referrals, confirming earlier findings,^10,14^ often within a short period. For example, patients with suicide attempts were five times more likely to have been previously referred to the PER because of suicide attempt, and for 18% of the patients, this happened within 1 month. Second, those previously referred for ideation/plan were at 66% higher risk of making the transition to suicide attempt, with 25% of patients making this transition within a month after previous referral. This was similar to the transition from depressed mood to suicide ideation/plan. These results suggest a clear transition trajectory through the STB spectrum in the PER, starting with depressive symptoms which may lead to suicide attempt in the emergency room. Third, a previous referral for suicide attempt was highly predictive of a consecutive referral to the PER for suicide attempt. Indeed, over one-third of patients previously referred for suicide attempt were referred again for the same reason and a quarter for suicide ideation/plan. Fourth, patients with substance use or psychotic symptoms were 52–55% less likely to return to the PER with any form of STB. Further analysis revealed substance use as being very persistent; hence, a previous referral for substance misuse was, more than anything, predictive of a referral to the PER again for substance misuse.

From the clinical perspective of the emergency room, our findings are a first step in enabling clinicians to identify those patients who carry the highest STB risk. Previous studies have shown that recent age-at-onset and controllability of ideation/plan are predictive of making a transition to suicide attempt.^1^ Although these are crucial elements in patients’ STB assessments, the rush of the emergency room often precludes clinicians from assessing such information reliably. Specifically, within a PER perspective, our data show that depressed mood precedes ideation/plan, and ideation/plan precedes suicide attempt, and that these transitions can occur within weeks. These are essential findings, stressing the need to take extreme care with such patients by, for instance, initiating a close treatment chain leading to altering the suicidal process.^7^ Clearly, investing more resources at these crucial moments in the suicidal process, in the PER, may drastically alter the outcome for these patients.

Patients with repeated STB in the PER often elicit negative feelings from non-psychiatric and psychiatric staff.^28^ They are referred to as patients who do not belong in emergency departments, as ‘hard to treat’ and difficult patients,^5^ or as patients that systematically overuse the PER. However, we found that this specific group of patients was the most at risk for STB. How these two elements fit together may depend on local policies in emergency departments, but from the viewpoint of suicide prevention, patients with depressive problems and/or suicide ideation/plan do not constitute specific counterindications for an emergency room. On the contrary, our data show that referrals to the PER can constitute crucial opportunities to intervene in the suicidal process.

Although studies in the general population and those based on clinical reports have highlighted temporal associations between substance misuse and STB (either ideation/plan or attempt),^29^ we could not find any evidence for such associations in our study. We found that those previously referred for substance misuse were about half as likely to be referred later with STB. We believe this was mainly due to the persistence of substance use problems and the particular context of the PER, where referral for substance misuse is prevalent (almost one-fourth of the population). We found a similar association between being referred earlier because of psychotic symptoms. This counterintuitive finding potentially challenges the traditional idea that psychosis and STB are closely related.^30^ However, it is important to keep in mind that our data concern snapshots at the PER. If patients manage to find professional support after the referral, they may no longer present at emergency facilities in case of crisis, as other options are known and available to them and their social network.

Being retrospective in nature, the present study had some notable strengths and limitations. The main strength of our study was its magnitude; overall, we covered a time frame of nearly 20 years, including approximately 25 000 PER referrals. Although our data dis not include all PER referrals at UZ Leuven in this time frame, we weighted the data to be representative of the general PER population. Moreover, we carefully imputed missing values to allow the most accurate estimates of uncertainty. By contrast, many PER studies are hampered by telescoping bias, i.e. patients’ experience that previous referrals to the PER would be recalled as more recent compared to their current referrel.^31^ Finally, we found that information on prior referrals and reason for referral hold predictive value for STB risk assessment. This is especially relevant for emergency rooms without psychiatric facilities, or facilities with limited resources, or those emergency room settings which prohibit staff from collecting detailed information regarding patients’ STB.

Our study also had notable limitations. First, the setting for this study, an academic teaching hospital emergency service, may limit the generalisability of our findings. In addition, the pathways through the STB spectrum were described from the perspective of the PER and may not be straightforwardly generalisable to the general population. Variables such as family history, hopelessness or history of NSSI were not systematically collected in a structured way and thus not considered.

Furthermore, as NSSI was not a distinct class of reasons for referral (NSSI was added to the semi-structured interview as a separate category in February 2020), we were unable to differentiate STBs from NSSI. Although it is unclear how NSSI was classified by clinicians throughout the two decades of data collection, additional analyses on records since 2020 revealed that only 1.63% (95% CI: 1.32–1.97%) of referrals to the PER were due to NSSI. Its clinical relevance is unequivocal – indeed, that is the reason NSSI was added as a separate category in 2020 – and it may be critical in prognosis and outcome. We assume that the main findings of the present study would not be substantially different if NSSI had been included as a separate category. In any case, the potentially specific transition course of NSSI will form the object of further study on the more recent referrals, as more data become available.

In the same vein, the linkage of suicide ideation and plan means that distinctions between an impulsive suicide attempt versus those planned were more difficult to identify in the data. Against the rush of a large emergency room, it is clinically difficult to make clear distinctions between ideation and plan. Similarly, data for completed suicides or estimates of such based upon the data were unavailable. From a clinical and scientific perspective, these should form the focus of further studies.

In addition, although missing data strategies such as multiple imputation techniques were implemented, imputations were valid to the extent that the missingness is at random. The last limitation is that our study focused on patients aged >17 years, potentially excluding the most vulnerable group for STB onset and transition.^32^

The main finding of the present study was that in the context of the PER, STB is a persistent condition, showing a clear progressive pattern in subsequent referrals; moreover, the time between these referrals can be extremely short. In addition, we established that at least for a subgroup of patients referred to the PER with depressive symptoms, there was a risk of transitioning to STB. From these findings, three clear recommendations follow. First, immediate risk assessment is essential to identify patients in the emergency room at risk of self-harm or suicide; staff training and education may ensure that emergency room staff will be able to recognise and manage patients who are suicidal when they are referred to the PER. Although we did not examine specific factors associated with persistence and progressive transitioning in this study, we did establish that they represent an unambiguous risk. Second, given that progressive transitions are likely and can occur in a short time frame, follow-up and continuity of care for these patients is essential.

Third, suicide prevention efforts in the emergency room should not be limited to building capacity for what is commonly referred to as ‘a suicide risk assessment’, that is, an assessment of suicide ideation, plans, attempts or NSSI.^33^ Given our findings, staff should also be aware that depressive symptoms (even in the absence of any STB) may lead to a STB referral within a month, indicating the need for continuity of care and more elaborate follow-up of these patients. Indeed, early intervention in STB is a key challenge, and safety planning should be initiated before the actual occurrence of STB. Unfortunately, so far, the potential of such early intervention has been limited because there are no specific factors that consistently precede STB.^34^ A more refined assessment that goes beyond merely establishing depressive symptoms may lead to the early identification of patients at risk of entering a pathway to STB. Potentially, such an assessment could, in combination with more recent developments in machine learning and artificial intelligence – with prediction models that can access the entire medical and psychiatric history of patients and allow for complicated non-linear relations and interactions – lead to decision support that could identify cases at risk with more sensitivity and specificity.

## Data Availability

All analysis scripts can be found on the Open Science Platform: (https://osf.io/hzg6v/), along with simulation data-sets.

## Footnotes

a. Given that the setting was a university hospital, there was a yearly change of doctors in training.
